# A comprehensive study of conditions of the biodegradation of a plastic additive 2,6-di-*tert*-butylphenol and proteomic changes in the degrader *Pseudomonas aeruginosa* san ai†

**DOI:** 10.1039/c9ra04298a

**Published:** 2019-07-30

**Authors:** Ana Medić, Ksenija Stojanović, Lidija Izrael-Živković, Vladimir Beškoski, Branka Lončarević, Saša Kazazić, Ivanka Karadžić

**Affiliations:** Department of Chemistry, Faculty of Medicine, University of Belgrade Višegradska 26 11000 Belgrade Serbia ivanka.karadzic@med.bg.ac.rs +381113607067; Faculty of Chemistry, University of Belgrade Studentski trg 12-16 11000 Belgrade Serbia; Institute of Chemistry, Technology and Metallurgy, Department of Chemistry Njegoševa 12 11000 Belgrade Serbia; Ruđer Bošković Institute Bijenička cesta 54 Zagreb Croatia

## Abstract

The *Pseudomonas aeruginosa* san ai strain was investigated for its capability to degrade the 2,6-di-*tert*-butylphenol (2,6-DTBP) plastic additive, a hazardous and toxic substance for aquatic life. This investigation was performed under different parameter values: 2,6-DTBP concentration, inoculum size, pH, and temperature. The GC-MS study showed that *P. aeruginosa* efficiently degraded 2,6-DTBP in the pH range of 5–8 at higher temperatures. Under exposure to 2,6-DTBP concentrations of 2, 10, and 100 mg L^−1^, the strain degraded by 100, 100, and 85%, respectively, for 7 days. Crude enzyme preparation from the biomass of *P. aeruginosa* san ai showed higher efficiency in 2,6-DTBP removal than that shown by whole microbial cells. Gene encoding for the enzymes involved in the degradation of aromatic compounds in *P. aeruginosa* san ai was identified. To complement the genomic data, a comparative proteomic study of *P. aeruginosa* san ai grown on 2,6-DTBP or sunflower oil was conducted by means of nanoLC-MS/MS. The presence of aromatic substances resulted in the upregulation of aromatic ring cleavage enzymes, whose activity was confirmed by enzymatic tests; therefore, it could be concluded that 2,6-DTBP might be degraded by *ortho*-ring cleavage. A comparative proteomics study of *P. aeruginosa* san ai indicated that the core molecular responses to aromatic substances can be summarized as the upregulation of proteins responsible for amino acid metabolism with emphasized glutamate metabolism and energy production with upregulated enzymes of glyoxylate bypass. *P. aeruginosa* san ai has a high capacity to efficiently degrade aromatic compounds, and therefore its whole cells or enzymes could be used in the treatment of contaminated areas.

## Abstract 2

The *Pseudomonas aeruginosa* san ai strain was investigated for its capability to degrade the 2,6-di-*tert*-butylphenol (2,6-DTBP) plastic additive, a hazardous and toxic substance for aquatic life.

## Graphical abstract

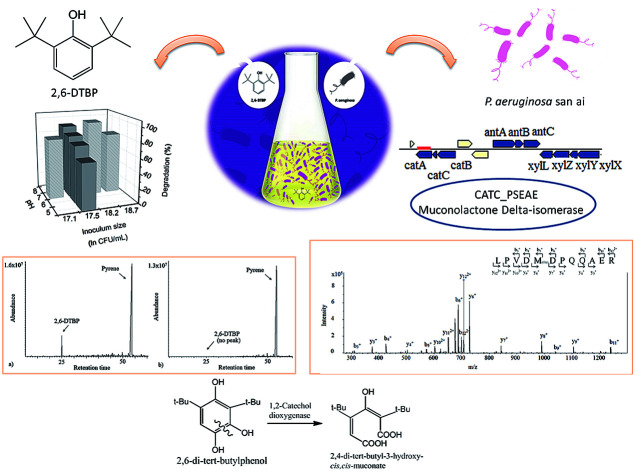

## 1. Introduction

The plastic additive 2,6-di-*tert*-butylphenol (2,6-DTBP) is an industrially important chemical that has been used as an antioxidant in plastics, as well as an oxidation inhibitor and stabilizer for fuel, oil, and gasoline.^1,2^ Antioxidants slow down the oxidation cycle and prevent the loss of strength, breakdown, or discoloration of plastics. Moreover, 2,6-DTBP is used as a synthetic intermediate for the production of higher-molecular-weight phenolic compounds. Due to the extensive use of 2,6-DTBP, the possible routes for its release into the natural environment, particularly in surface water, are manufacturing processes and leaching from the final products. Because of concerns regarding the widespread use of microplastics and their effects on the aquatic environments and their biota,^3^ 2,6-DTBP could become a serious issue for aquatic animals; this is because 2,6-DTBP has shown highly acute toxicity to crustacean (*Gammarus fasciatus*), fathead minnow (*Pimephales promelas*), water flea (*Daphnia magna*), and zebrafish (*Brachydanio rerio*) with LC_50_ values of 0.80, 1.1, 0.45, and 10 mg L^−1^, respectively.^1^ In the case of human exposure, skin and eye irritations have been reported.^1^

As chemical decomposition is usually complex and uneconomical, biodegradation is considered to be a promising alternative to remove 2,6-DTBP from polluted areas. The degradation of environmental pollutants—particularly organic compounds (hydrocarbons, including polycyclic aromatic hydrocarbons (PAHs)), phenols, and its derivatives—by certain naturally occurring microorganisms have been considered to be a safe, efficient, cost-effective, and environmentally friendly technology for treating hazardous substances.^4,5^ However, the biological remediation of aromatic substances is particularly challenging as aromatic rings are highly stable against microbial degradation. Although microorganisms that can degrade, transform, or accumulate toxic organic compounds are not widely distributed in nature, a certain number of bacterial species (*e.g.*, *Bacillus stearothermophilus*, *Bacillus laterosporus*, *Alcaligenes eutrophus*, *Rhodococcus erythropolis*, and *Rhodococcus* sp., particularly genus *Pseudomonas*) have been described to degrade a wide variety of aromatic compounds, although at lower pollutant concentrations in most cases.^6^ The natural abundance of microorganisms with multidegradative capacity, which may play an important role in the removal of heterogeneous contamination, is well reviewed.^7^ The *Pseudomonas* genus is known for its ability to utilize diverse aromatic compounds as the sole C source.^8,9^ The degradation and utilization of alkylphenols have been reported for several *Pseudomonas* strains, including *Pseudomonas* sp. KL28,^10^*Pseudomonas putida* MT4,^11^ and *Pseudomonas veronii* INA06.^12^ Only two *Pseudomonas* strains, namely, *Pseudomonas azelaica*^13^ and *Pseudomonas* sp. MS-1, have been reported to degrade *ortho*-monosubstituted alkylphenols,^14^ whereas *Pseudomonas putida* TX2 and *Pseudomonas* sp. TX-1 can degrade *para*-monosubstituted alkylphenols.^15^ There is only one publication on the biodegradation of *ortho*-disubstituted alkylphenols.^16^

Recent advances in high-throughput genomics and proteomics techniques have opened up new opportunities for understanding the molecular mechanism of biodegradation.^17^ A specific cellular response during growth on different C sources, including organic pollutants, affects the proteome of the microorganism. An insight into such mechanisms associated with organic pollutant removal, as well as the dynamics of synthesis of certain proteins, can address the gap involving the fundamental understanding of the molecular mechanisms of the adaptation of microorganism, strategies to better control production of its worthwhile metabolites, and apply them toward environmental protection. Simultaneously, differential up- or downregulation of proteins/enzymes under the distinct conditions of growth could serve toward the environmental biomonitoring of stress induced by pollutants in the natural microbial communities and for biomarker measurement. Until now, published data have revealed considerable changes in microbial proteomes during aromatics degradation in comparison to those in natural C sources.^18–25^ Recently, a global proteomics approach was successfully used to explore phenanthrene catabolic pathways in *Arthrobacter phenanthrenovorans* exposed to phenanthrene,^20^ implying an overproduction of enzymes of degradation and their strong downregulation when the culture is grown on aromatic and natural substrates, respectively. Further, global, ge1-based 1-DE in combination with tandem MS proteomics approach was considered to be a sensitive method for mapping the enzymes involved in the phenol degradation of pseudomonad, an indigenous soil.^25^ No proteomics studies of microbial response toward alkylphenols have been conducted so far.

The β-ketoadipate pathway is an aryl-ring degradation pathway for the conversion of hazardous aromatic pollutants into nontoxic metabolites, which are widely distributed in bacteria (including *Pseudomonas*).^5^ This pathway mediates the *ortho*-cleavage of dihydroxy-substituted arenes through two distinct branches—catechol and protocatechuate. Most of the published strains degrade alkylphenols *via* alkylcatechols using the *ortho*- or *meta*-cleavage of the aromatic ring.^14,15^ The biodegradation of the *ortho*-disubstituted derivatives of alkylphenols has not been studied in detail yet.

*Pseudomonas aeruginosa*, well known for its ability to grow in diverse environments and with different C sources, has shown promising potential in the remediation of sites polluted by hardly biodegradable organic chemicals.^26^ The main objective of this study involves the estimation of the capability of *P*. *aeruginosa* san ai, a culturable environmental isolate from mineral cutting oil,^27^ to degrade the *ortho*-disubstituted alkylphenol—2,6-DTBP—under different parameters, namely, 2,6-DTBP concentration, inoculum size, pH, and temperature. The successful identification of catabolic pathways using the proteomic approach^20,25^ has encouraged us to perform comparative proteomics to explore the biodegradation pathway along with the metabolic adaptation of the microorganism grown on 2,6-DTBP and sunflower oil as the sole C source. The comparative proteomics approach based on nanoliquid chromatography and tandem mass spectrometry (nanoLC-MS/MS) coupled with bioinformatics was applied to identify proteins. The proteomic results of the biodegradation of aromatic compound by *P. aeruginosa* san ai were validated by the enzymic approach.

## 2. Material and methods

### Chemicals

2,6-DTBP and the organic solvents were purchased from Sigma Aldrich (St. Louis, MO, USA) with purity above 99% and of the HPLC grade. All the other chemicals used in this study were of the pro-analysis grade purchased from Merck (Darmstadt, Germany).

### Media and growth conditions

The microorganisms used for 2,6-DTBP degradation were *Pseudomonas aeruginosa* san ai, a strain isolated from industrial mineral metal-cutting oil.^27^ The strain was deposited in National Collection of Agricultural and Industrial Microorganisms (NCAIM), Faculty of Food Sciences, Corvinus University of Budapest, Budapest, Hungary, labeled as NCAIM B.001380 and ISS WDCM 375 (Institute of Soil Science, Belgrade, Serbia) in the public collection with accession number ISS 619.

*P. aeruginosa* san ai was activated on nutrient agar (Torlak, Belgrade, Serbia) at 30 °C for 24 h and transferred to a 500 mL Erlenmeyer flask, containing 100 mL of minimal salt medium (MSM) supplemented with different sources of carbon and energy, to achieve an initial colony-forming unit (CFU) value of approximately 4 × 10^7^. 2,6-DTBP, sodium benzoate, and sunflower oil were used as the C sources. Moreover, the strain was grown in a complex Luria–Bertani (LB) broth. MSM comprised the following components (per liter): 5 mL phosphate-buffered solution (PBS; KH_2_PO_4_ 8.5 g L^−1^, K_2_HPO_4_ 19.71 g L^−1^, Na_2_HPO_4_·2H_2_O 16.61 g L^−1^, and NH_4_Cl 5 g L^−1^); 3 mL MgSO_4_·7H_2_O solution (46.125 g L^−1^); 1 mL FeCl_3_ solution (0.25 g L^−1^); 1 mL CaCl_2_·2H_2_O solution (48.21 g L^−1^); 1 mL trace element solution (MnSO_4_·4H_2_O 0.05 g L^−1^, (NH_4_)_6_Mo_7_O_24_·4H_2_O 0.0347 g L^−1^, and ZnSO_4_·7H_2_O 0.07 g L^−1^)^28^ was sterilized by a Millipore filtration system (Merck Millipore, USA) using a membrane filter (0.45 μm). The pH of this medium was maintained between 7.2 and 7.4. An appropriate volume of the stock solution of 2,6-DTBP in *n*-hexane was injected into sterile 500 mL Erlenmeyer flasks to obtain the desired final concentration and then hexane was allowed to evaporate under the airflow. After forming a thin film of 2,6-DTBP at the bottom of the flask, 100 mL sterilized MSM was added to each flask.^29^ Sodium benzoate as the C source was dissolved in water and added to MSM. The flasks were incubated at 30 °C for 7 days and shaken at 150 cycles per min using a horizontal shaker (Kuhner, Basel, Switzerland). MSM with appropriate concentrations of C sources without *P. aeruginosa* san ai was used as the control for nonbiological degradation.

Bacterial growth was monitored as a change in the optical density (OD) at 580 nm, measured on an ultraviolet-visible spectrophotometer (UV-2600, Shimadzu Kyoto, Japan) using a sterile non-inoculated medium as the reference.^30^ The bacterial concentration was estimated to be 3 × 10^8^ cells per mL, which corresponded to an absorbance value of 0.47 at 580 nm.

### Effects of initial concentration of 2,6-DTBP, pH, temperature, and inoculum size on biodegradation

These effects were examined using MSM (pH 7.2) supplemented with 100 mg L^−1^ 2,6-DTBP inoculated with 4 × 10^7^ CFU per mL and shaken at 150 cycles per min using a horizontal shaker at 30 °C for 7 days, unless otherwise indicated. A sterile medium with 2,6-DTBP as the control of chemical decomposition and inoculated sterile MSM without 2,6-DTBP as the control of microbial growth were designed. All the experiments were performed in three independent replicates and the average values are given in this study. The differences in the results between the replicates in all the experiments did not exceed 1.5%.

Several concentrations of 2,6-DTBP, namely, 2, 10, 100, and 400 mg L^−1^, were added to MSM to investigate the effects of initial concentration.

The effect of pH on the biodegradation efficiency was assessed by individually modifying the pH of MSM to 5, 6, 7, and 8. The pH was adjusted before membrane filtration using HCl or NaOH solutions at a concentration of 0.1 mol L^−1^.

The influence of temperature on 2,6-DTBP biodegradation was investigated at four different values: 22, 30, 37, and 43 °C.

To study the effect of inoculum size, cell suspension of *P. aeruginosa* san ai was added to the medium to achieve initial CFU per mL values of 2.6 × 10^7^, 8.3 × 10^7^, and 1.4 × 10^8^.

### Microbial adherence to *n*-hexadecane (MATH) test

Cell hydrophobicity was evaluated *via* the MATH test.^31^ Two milliliters of bacterial suspension in distilled water at OD_580_ = 0.5 was overlaid with 0.5, 1, and 1.5% (v/v) of *n*-hexadecane. After vortexing for 1 min, the phases were permitted to separate for 15 min. The absorbance at 580 nm of the aqueous phase was then measured. The results were calculated using the following equation: [(*A*_0_ − *A*)/*A*_0_] × 100, where *A*_0_ and A represent the initial and final ODs of the aqueous phase, respectively.

### Gas chromatography-mass spectrometry (GC-MS) analysis of 2,6-DTBP and its degradation products

The entire flask culture was extracted by *n*-hexane for three times. The extracts were dehydrated with anhydrous Na_2_SO_4_ and the solvents were evaporated under reduced pressure by a rotary evaporator (Senco GG17, Shanghai, China). The extraction residues were dissolved in *n*-hexane and analyzed by GC-MS. The gas chromatograph Agilent 7890A GC (HP5-MS capillary column; 30 m × 0.25 mm; film thickness: 0.25 μm; He carrier gas: 1.5 cm^3^ min^−1^) coupled to an Agilent 5975C mass selective detector (70 eV) was used. The column was heated from 80 to 300 °C at a rate of 2 °C min^−1^ and maintained at 300 °C for 20 min; thereafter, it was heated from 300 to 310 °C at a rate of 10 °C min^−1^ and maintained at 310 °C for 1 min. The injector temperature was 250 °C. The spectrometer was operated in the electrospray ionization (EI) mode over a scan range from *m*/*z* 45 to 550. The individual peaks of 2,6-DTBP and its degradation products were identified on the basis of the mass spectra (library: NIST11). The quantification of the compounds in the standard series, controls (sterile medium with 2,6-DTBP), and inoculated samples (sterile medium supplemented with 2,6-DTBP and *P. aeruginosa* san ai) were performed by an integration of the peak areas (software: GCMS Data Analysis). For the calculations, a standard series of commercial 2,6-DTBP solutions in *n*-hexane covering the range of 2,6-DTBP concentrations from 2 to 400 mg L^−1^ (2, 5, 10, 50, 100, 200, and 400 mg L^−1^), as used in the study, were prepared and analyzed under the same GC-MS conditions as those for the samples. The ratio of the peak areas and the corresponding concentrations were linearly dependent with very high correlation (*r*^2^ = 0.994). Moreover, in order to verify the stability of 2,6-DTBP, two identical standard series, as mentioned above, were prepared and analyzed by GC-MS. The first standard series was analyzed after preparation, and the second one after 7 days from preparation (the time of experiments); they were exposed to the same temperature (30 °C) during this period. The obtained results from the GC-MS analysis showed no change in 2,6-DTBP concentrations in the solutions of the standard series after 7 days. Furthermore, a comparison of the GC-MS results of the standard series and control samples, having the same concentration of 2,6-DTBP, indicated that the losses of the target compound in the controls were less than 2%, confirming the accuracy of the experiments. The efficiency of degradation is determined based on the concentrations of the target compound in the control and inoculated samples, and it is expressed in percentage.

### Crude enzyme extract preparation

Total proteins were isolated from the biomass grown in MSM supplemented with different C sources in the early stationary phase. Biomass was collected by centrifugation, frozen, and homogenized in two volumes of buffer A (50 mM Tris buffer at pH 7.5 supplemented with 0.1 mM phenylmethylsulfonyl fluoride (PMSF) and 0.5 mM 1,4-dithiothreitol (DTT)) in a glass Teflon homogenizer at 4 °C. The resulting homogenates were ultracentrifuged for 2 h at 100 000*g* at 4 °C using Beckman Coulter L5-65 with SW 28 rotor (Beckman, Indianapolis, USA). The supernatants were used for proteomics and enzyme assays after the determination of protein content by the Bradford's method.^32^

### Proteomic analysis

Crude protein extracts obtained from the biomass grown on MSM supplemented with 100 mg L^−1^ 2,6-DTBP or 100 μL L^−1^ sunflower oil were used for proteomics analysis. The crude protein extract was separated by sodium dodecyl sulfate (SDS) polyacrylamide gel electrophoresis (PAGE) using 4–15% gradient acrylamide gels (Criterion™ TGX™ Precast Gels, Bio-Rad) according to the manufacturer's instructions. Staining was carried out with Coomassie Brilliant Blue R-250. Twelve successive bands from the gel were cut and washed with 50 mM ammonium bicarbonate and 100% acetonitrile (ACN) for 15 min on the thermomixer (Digital Shaking Drybath, Thermo Fisher Scientific, USA). After volume reduction by a vacuum concentrator (Eppendorf Concentrator 5301, Hamburg, Germany), proteins were digested by trypsin (Promega, Madison, USA) dissolved in 50 mM ammonium bicarbonate to the final concentration of 0.02 μg μL^−1^. Digestion was performed overnight at room temperature on the thermomixer. The eluate was saved and gel-washed with 5% ACN/0.1% formic acid (FA) and 50% ACN/0.1% FA for 15 min on the thermomixer. The washes were combined and concentrated to near dryness in the vacuum concentrator.

Peptide digests were analyzed by EI in the positive mode on an ion-trap instrument called Amazon Speed (Bruker, Bremen, Germany) using a captive spray source. Two analytical replicates of every sample were conducted. Peptides were separated by nanoflow HPLC (NanoAdvance, Bruker, Bremen, Germany). UHPLC Nanotrap (i.d.: 100 μm; length: 25 mm) packed with 200 A C18 stationary phase (5 μm, C18AQ, Michrom) was used for peptide trapping. Analytical columns (100 μm × 100 mm long) packed with 130 A C18 stationary phase (1.7 μm, ACQUITY, UPLC, M-Class, Waters) were coupled to the mass spectrometer (MS). Peptide mixtures obtained after tryptic digestion were applied to the precolumn at a flow rate of 5 μL min^−1^ in 2% (v/v) ACN with 0.1% (v/v) FA. The peptides were eluted by a linear gradient of A (water, 0.1% FA) and B (ACN, 0.1% FA) as follows. 0 min: A (98%), B (2%); 50 min: A (5%), B (95%); 50–55 min: A (5%) and B (95%) at a flow rate of 400 nL min^−1^. The ion source conditions were optimized with a calibration solution according to the instrument provider's instructions. All the MS survey scans were performed from *m*/*z* 400 to 1400 with enhanced resolution. The data analysis was undertaken by the selection of the five most abundant precursors rejecting singly charged ions.

The tandem mass spectral data were automatically acquired and processed using Hystar 3.2 and Data Analysis 4.2 software (Bruker, Germany). The deconvoluted MGF spectra were searched by using the Mascot database search tool (version 2.3.02). The mascot search parameters were set as follows. Taxonomy: other proteobacteria; enzyme: trypsin; missed cleavages: 2; modifications, cysteine carbamidomethylation and methionine oxidation (variable); precursor mass tolerance: 1.2 Da; MS/MS mass tolerance: 0.6 Da; ions with monoisotopic *m*/*z* value and +2 and +3 charge states. Only the top hits in the compounds' list with a significance threshold of *p* < 0.01 were considered.

### Enzyme assays

The activities of catechol 1,2-dioxygenase (C12O) and catechol 2,3-dioxygenase (C23O) were spectrophotometrically measured at 260 and 375 nm, respectively, using UV-2600. The formation of 2-hydroxymuconic semialdehyde, *cis*,*cis*-muconic acid, and their derivatives was measured by slightly modifying the procedures proposed by Briganti *et al.*^33^ and Mahiudddin *et al.*^34^ The reaction mixture for C12O activity determination comprised the following: 1 mL of 50 mM PBS at pH 8.0, 0.1 mL of 100 mM DTT, and 0.1 mL of cell homogenate obtained from biomass grown on 2,6-DTBP or sodium benzoate. The reaction was started by the addition of 0.1 mL of 1 mM substrate (catechol, 2,6-DTBP, and sodium benzoate). The reaction mixture for C23O activity determination comprised the following: 1 mL of 100 mM PBS at pH 8.0, 0.1 mL of cell homogenate, and 0.1 mL of 100 mM substrate (catechol, 2,6-DTBP, and sodium benzoate). One unit of activity was defined as the amount of enzymes producing 1 μmol each of *cis*,*cis*-muconate (*ε*_260_ = 1.6 × 10^4^ mol^−1^ cm^−1^),^34^ 2-hydroxymuconic acid (*ε*_290_ = 1.2 × 10^4^ mol^−1^ cm^−1^),^35^ and 2-hydroxymuconic semialdehyde (*ε*_375_ = 4.4 × 10^4^ mol^−1^ cm^−1^)^25^ per min at 25 °C. The specific activity is calculated as the enzyme activity per mg of protein.

The oxygenolytic activity of the homogenate was measured by amperometrically recording the O_2_ consumption by a Clark electrode. The experiment was performed at 25 °C in a 5 mL chamber containing 4 mL of 100 mM PBS at pH 8.0, 0.4 mL substrate (2,6-DTBP), and 0.4 mL crude protein extract.^36,37^

The activity of catalase (CAT) was assayed by the measurement of H_2_O_2_ substrate remaining after the action of CAT.^38^ One unit (U) of CAT activity was defined as 1 μmol of H_2_O_2_ degraded into O_2_ and H_2_O per min of reaction. The specific activity is calculated as the enzyme activity per mg of protein.

The activity of superoxide dismutase (SOD) was determined according to the method based on the oxidation of adrenalin previously described by Sun and Zigman.^39^ The change in absorbance was determined at 340 nm. One unit (U) of SOD activity is the amount of enzyme that causes a change in absorbance (Δ*A*/min) of 0.001 under the test conditions. The specific activity was calculated as the enzyme activity per mg of protein.

### Enzymatic degradation

Crude protein extracts obtained from biomass grown in MSM with 2,6-DTBP for 48 h were used to investigate the enzymatic degradation of 2,6-DTBP. The experiment was performed in lightproof 500 mL bottles (Duran, Wertheim, Germany) containing 50 mL of 100 mM PBS at pH 8.0, supplemented with 4 mL substrate (2,6-DTBP) at a concentration of 10 mg L^−1^, and 4 mL of the crude protein extracts; the mixture was constantly stirred (150 rpm) with a magnetic stirrer (Heidolph, Schwabach, Germany) at 29 + 2 °C for 4 days. The remaining amount of 2,6-DTBP was estimated by the GC-MS analysis, as described. Two controls were designed: crude extracts without substance and only substance without cell extracts in PBS at pH 8.0.

### Respiration analysis

The respiration activity of *P. aeruginosa* san ai exposed to 2,6-DTBP in MSM was measured using a twelve-channel Micro-Oxymax® respirometer (Columbus Instruments, Columbus, USA) connected to a PC. The experiments were performed in Micro-Oxymax lightproof 500 mL bottles (Duran, Wertheim, Germany) containing 100 mL MSM supplemented with different concentrations of 2,6-DTBP and constantly stirred (150 rpm) with a magnetic stirrer (Heidolph, Schwabach, Germany) at different temperatures for 4 days. The respiration rates (μL min^−1^) as well as the cumulative O_2_ consumed and CO_2_ produced (μL) were determined. Cell respiration was measured every 300 min for 4 days. A sterile medium with 2,6-DTBP and inoculated sterile MSM without 2,6-DTBP were used as the controls. All the experiments were performed in triplicate. The obtained data were evaluated by Micro-Oxymax software.

## 3. Results and discussion

**Fig. 1:**
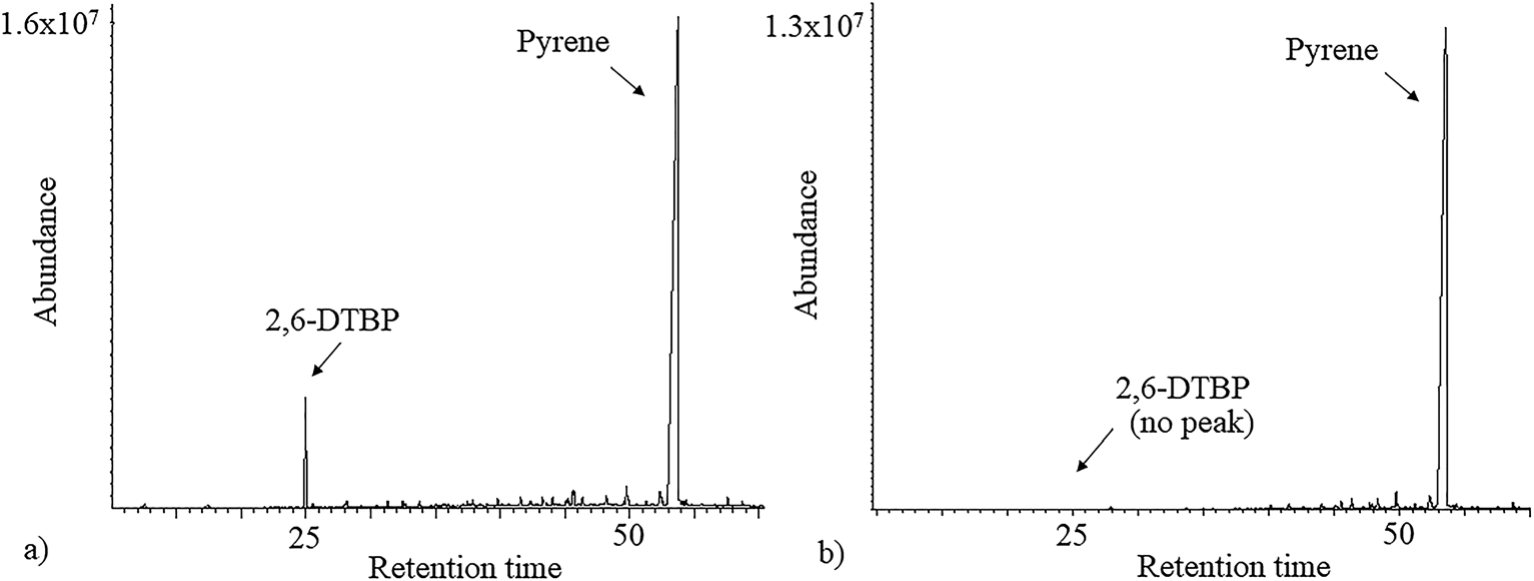
Total ion chromatogram (TIC) of *n*-hexane extracts of (a) control and (b) inoculated culture broth supplemented with pyrene.

As 2,6-DTBP is particularly harmful to aquatic life, all the experiments of biodegradation by *P. aeruginosa* san ai were done in MSM, which mimics an aquatic environment, as the growth medium. The direct correlation between the microbial surface hydrophobicity and its potential to degrade nonpolar organic polymers has already been reported;^40^ the cell surface of *P. aeruginosa* san ai was preliminarily determined. The degree of cell hydrophobicity of 29% was obtained by the MATH assay, which indicated moderate hydrophobicity^41^ of *P. aeruginosa* san ai and suggested its potential toward the biodegradation of different organic compounds. Following this idea, the degradation effects of several aliphatic and aromatic hydrocarbons (*n*-hexadecane, *n*-nonadecane, fluorene, phenanthrene, and pyrene) by *P. aeruginosa* san ai were tested. Interestingly, in chromatograms of the investigated substances, a peak corresponding to 2,6-DTBP, originating from the plastic used in this experiment, was noticed; surprisingly, this peak disappeared relatively rapidly during microbial growth (Fig. 1), which led us to the idea that the microorganism preferred to use 2,6-DTBP as the C source. Indeed, our preliminary experiments clearly demonstrated that *P. aeruginosa* san ai could remove 2 mg L^−1^ of 2,6-DTBP within 7 days. The effects of various conditions including initial 2,6-DTBP concentration, pH, and temperature on the degradative capabilities of *P. aeruginosa* san ai were further investigated.

### Biodegradation efficiency of *P. aeruginosa* san ai

#### Effect of initial substrate concentration

**Fig. 2:**
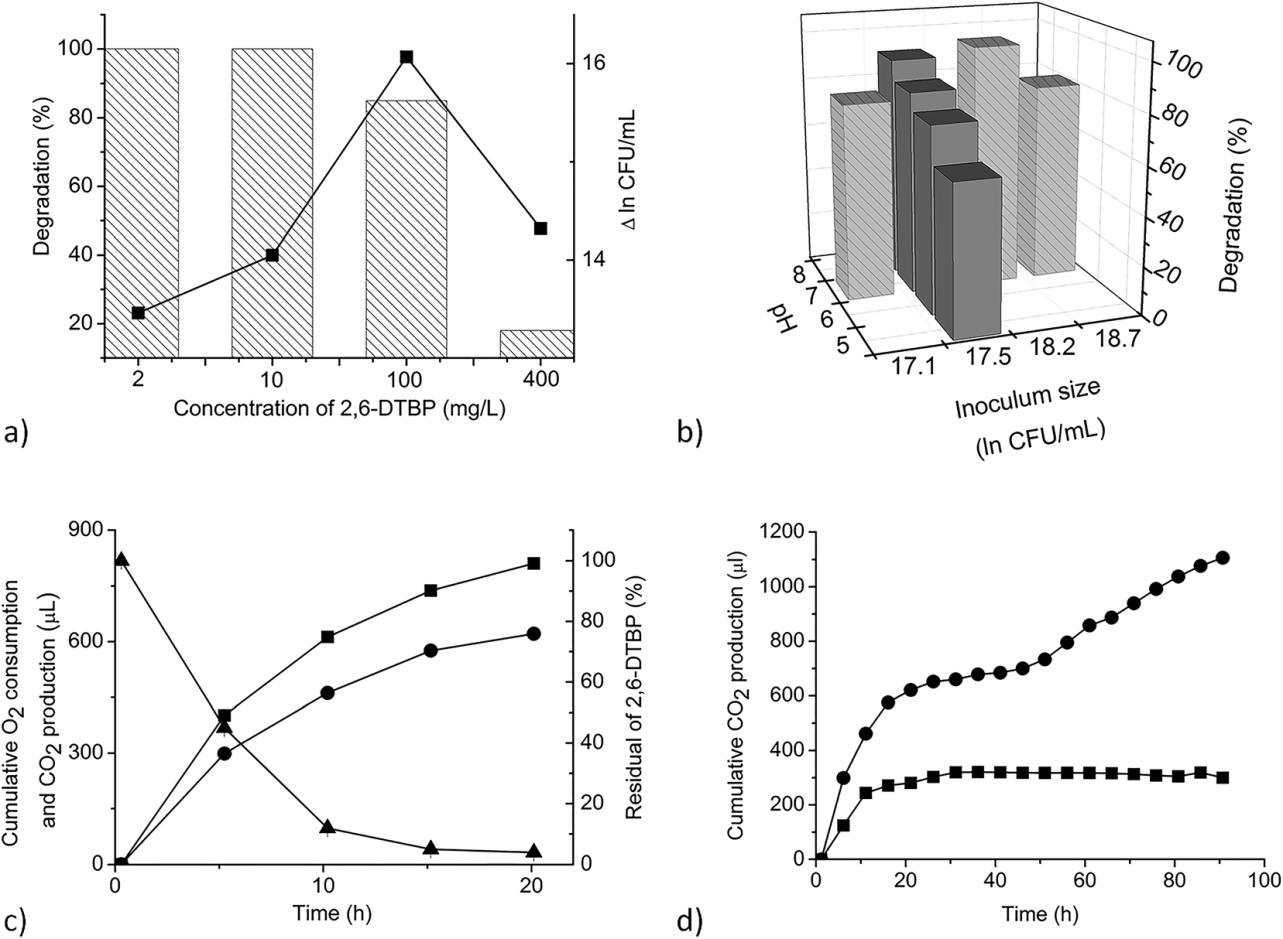
Effects of different factors and dynamics of 2,6-DTBP degradation. (a) Effect of initial substrate and intensity of growth of *P. aeruginosa* san ai. Efficiency of degradation was measured after 7 days. Biomass increase in the early stationary phase when the culture reached the maximum growth is shown (square) (Δ ln CFU per mL represents the difference between CFU per mL in the early stationary phase and that at time zero). (b) Effects of inoculum size and pH (C_2,6-DTBP_ = 100 mg L^−1^). Dark gray histograms indicate the effect of pH on degradation efficiency. Light gray histograms with textures show the effect of inoculum size on degradation efficiency. (c) Dynamics of the degradation of 2,6-DTBP (concentration: 4 mg L^−1^) (triangle), cumulative O_2_ consumption (square), and cumulative CO_2_ production (circle) of *P. aeruginosa* san ai grown at 30 °C (controls were subtracted). (d) Cumulative CO_2_ production during the growth of *P. aeruginosa* san ai on 2,6-DTBP (concentration: 10 mg L^−1^) at 25 °C (square) and 35 °C (circle) (controls were subtracted). Each data point represents the mean of three replicate samples. Some error bars are not visible because they are shorter than the symbol size.

MSM supplemented with increasing concentrations of 2,6-DTBP (2, 10, 100, and 400 mg L^−1^) as the sole C source was inoculated with the cell suspension of *P. aeruginosa* san ai at a starting CFU per mL value of approximately 4 × 10^7^ and cultivated for 7 days; thereafter, 100, 100, 85, and 18% of 2,6-DTBP was degraded, respectively (Fig. 2a). As shown in Fig. 2, the CFU per mL values increased as the concentration of 2,6-DTBP increased, reaching the maximum at the concentration of 100 mg L^−1^. Therefore, 100 mg L^−1^ was selected as the optimal concentration for further investigations on the effects of temperature and pH on the growth and degradation processes. The initial substrate concentration has a strong effect on the biodegradation efficiency. As shown in Fig. 2a, while lower concentrations of 2,6-DTBP (2 and 10 mg L^−1^) were completely degraded, the degradation of higher concentrations of 2,6-DTBP (100 and 400 mg L^−1^) decreased gradually. Namely, 85% of 2,6-DTBP of the initial concentration of 100 mg L^−1^ was successfully removed from the liquid medium within 7 days, implying a high capacity of the strain to not only survive but efficiently degrade the toxic compound from the contaminated areas, particularly from water (2,6-DTBP solubility: 4.0 mg L^−1^).^42^ On the other hand, the exposure to a higher concentration of 400 mg L^−1^ of 2,6-DTBP caused lower removal efficiency, which indicated the presence of toxic intermediates (ESI†).

#### Effect of inoculum size

In order to determine the influence of the inoculum size on the degradation of 2,6-DTBP, in addition to the CFU per mL value of 4.0 × 10^7^ (described above), initial CFU per mL values of 2.6 × 10^7^, 8.3 × 10^7^, and 1.4 × 10^8^ were analyzed, and degradations of 80, 99, and 81% of 2,6-DTBP were achieved, respectively (Fig. 2b). The percentage of degradation did not fully correlate with the inoculum size (Fig. 2b). As a matter of fact, inhibition effects with increased inoculum size were reported for bisphenol A degradation by *Achromobacter xylosoxidans*^43^ and *Sphingomonas* sp.,^44^ while the inoculum size was found to correlate with the efficiency of 2,6-DTBP degradation by a bacterium named F-3-4.^16^

#### Effect of pH

The pH values of MSM supplemented with 100 mg L^−1^ of 2,6-DTBP were adjusted to 5.0, 6.0, 7.0, and 8.0. As shown in Fig. 2b, the *P. aeruginosa* strain could efficiently degrade 2,6-DTBP in a wide range of pH values (from 5.0 to 8.0) with degradations at pH 5, 6, 7, and 8 of 61, 76, 83, and 91%, respectively, after 7 days. The maximum 2,6-DTBP degradation was observed at pH 8.0. Zhang *et al.*^16^ determined the optimum pH to be 7. The efficient degradation of 2,6-DTBP in a broad range of pH values implies the existence of pH-extensive enzymatic machinery for degradation, which is in good agreement with the previous data related to the broad pH stability of enzymes from the studied strain.^28,45^

#### Effect of temperature

Considering the lower metabolic activity of the strain at 25 °C (Fig. 2d), the degradation of 100 mg L^−1^ of 2,6-DTBP by *P. aeruginosa* san ai was investigated at temperatures of 22, 30, 37, and 43 °C. The percentage of 2,6-DTBP removal increased with an increase in temperature from 22 to 37 °C as follows: 45, 85, and 99%, but significantly decreased at 43 °C (25%) after 7 days. As compared to the referred efficiency of 2,6-DTBP removal by the F-3-4 strain,^16^*P. aeruginosa* san ai yielded better capability in a broad temperature range. According to the literature, with an increase in temperature, the biodegradation of phenolic compounds increased as the microbial growth and substrate solubility improved. However, microbial growth rates increased with an increase in temperature from 10 to 30 °C; the growth rates did not significantly change between 35 and 40 °C when the denaturation of proteins progressed.^46^ At the same time, the solubility of the phenolic substrate increased with increasing temperature. A higher production of CO_2_ at 35 °C supported the idea of accelerating the reactions by 2.5–3 times every 10° (Fig. 2d). As a matter of fact, intensified metabolism and mineralization at a higher temperature occurred. The crucial reason for the improved degradation is the increased solubility of 2,6-DTBP at a higher temperature, which, consequently, increases substrate availability for the microbial cells.

### Dynamic of 2,6-DTBP biodegradation

The dynamics of 2,6-DTBP biodegradation was monitored during *P. aeruginosa* san ai growth on 4 mg L^−1^ 2,6-DTBP, a concentration that is completely soluble in water.^43^ 2,6-DTBP was rapidly removed from the medium, intensively consuming O_2_ and releasing CO_2_, as shown in Fig. 2c. The most intensive respiration occurred in the first 5 h, with cumulative O_2_ consumption of 402 μL and cumulative CO_2_ production of 293 μL. At the same time, 50% 2,6-DTBP as the sole C source was mineralized. Within the first 10 h, more than 90% 2,6-DTBP was degraded. The cumulative production of CO_2_ reached the maximum after 20 h when complete 2,6-DTBP was exhausted. *P. aeruginosa* san ai quickly, within 20 h, removed 2,6-DTBP from the solution (concentration: 4 mg L^−1^), clearly demonstrating promising potential for the aerobic biodegradation of 2,6-DTBP (Fig. 2c). In fact, biodegradation by *P. aeruginosa* san ai was incomparably more efficient than anaerobic degradation by an activated sludge, where a conversion of 3.5% at the starting concentration of 0.05 mg L^−1^ 2,6-DTBP to CO_2_ was reported over 5 days.^47^

As biodegradation under different conditions of initial concentrations, pH, inoculums size, and temperature revealed the promising potential of *P. aeruginosa* san ai to degrade 2,6-DTBP, we further endeavored to study the genome and changes in the proteome of the strain, as well as to elucidate the metabolic pathways that contribute toward the survival under exposure to 2,6-DTBP.

### Genomic and proteomic insights into aromatic biodegradation

To explore the 2,6-DTBP catabolic pathways, genomic and proteomic analyses were applied. A collection of genes encoding to the proteins involved in the catabolism of aromatic compounds were identified in *P. aeruginosa* san ai (Genbank: BioProject PRJNA19571; accession number: JMKR00000000).^48^

**Fig. 3:**
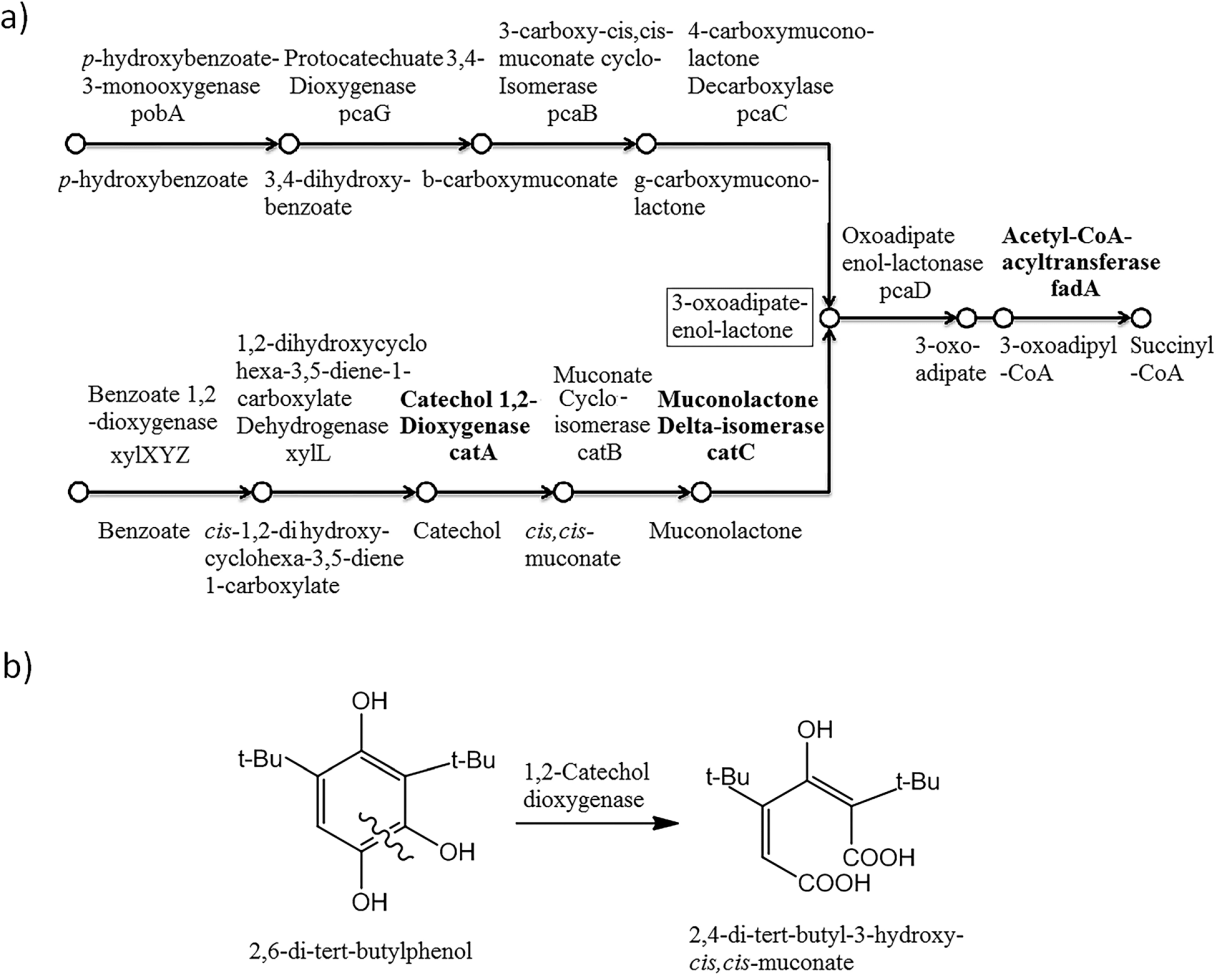
β-ketoadipate pathway in *P. aeruginosa* san ai. Compounds, genes, and enzymes are indicated. (a) Gene and coding protein, respectively, are as follows. pobA: *p*-hydroxybenzoate-3-monooxygenase; pcaG: protocatechuate 3,4-dioxygenase; pcaB: 3-carboxy-*cis*,*cis*-muconate cycloisomerase; pcaC: 4-carboxymuconolactone-decarboxylase; pcaD: oxoadipate-enol-lactonase; catA: catechol 1,2-dioxygenase; catC: muconolactone δ-isomerase; catB: muconate cycloisomerase; xylL: 1,2-dihydroxycyclohexa-3,5-diene-1-carboxylate dehydrogenase; xylXYZ: benzoate/toluate 1,2-dioxygenase. (b) Proposed *ortho*-degradation of 2,6-DTBP with 2,4-di-*tert*-butyl-3-hydroxy-*cis*,*cis*-muconic acid as the degradation product.

The biodegradation of alkylphenols by several *Pseudomonas* strains indicated that both C12O and C23O had wide substrate specificity for a number of alkylcatechols, which are the initial products of alkylphenol oxidation.^15^ With respect to these results, the genome of *P. aeruginosa* san ai was analyzed by KEGG and the presence of gene encoding to proteins involved in the catabolism of aromatic compounds (Fig. 3a) was detected in the K260DRAFT_scf7180000000062_quiver.15 and K260DRAFT_scf7180000000064_quiver.13 scaffolds. In particular, all the gene coding for proteins involved in the β-ketoadipate *ortho*-degradation pathway—catABC of catechol branch and pcaBCDG of protocatechuate branch—have been identified (Fig. 3a). Genomic analysis clearly indicated the potential of *P. aeruginosa* san ai for the degradation of aromatics through the *ortho*-pathway. To validate the genomic data, we used comparative proteomics to identify the proteins involved in the catabolism of 2,6-DTBP by *P. aeruginosa* san ai when grown on 2,6-DTBP *versus* sunflower oil as the C source.

**Fig. 4:**
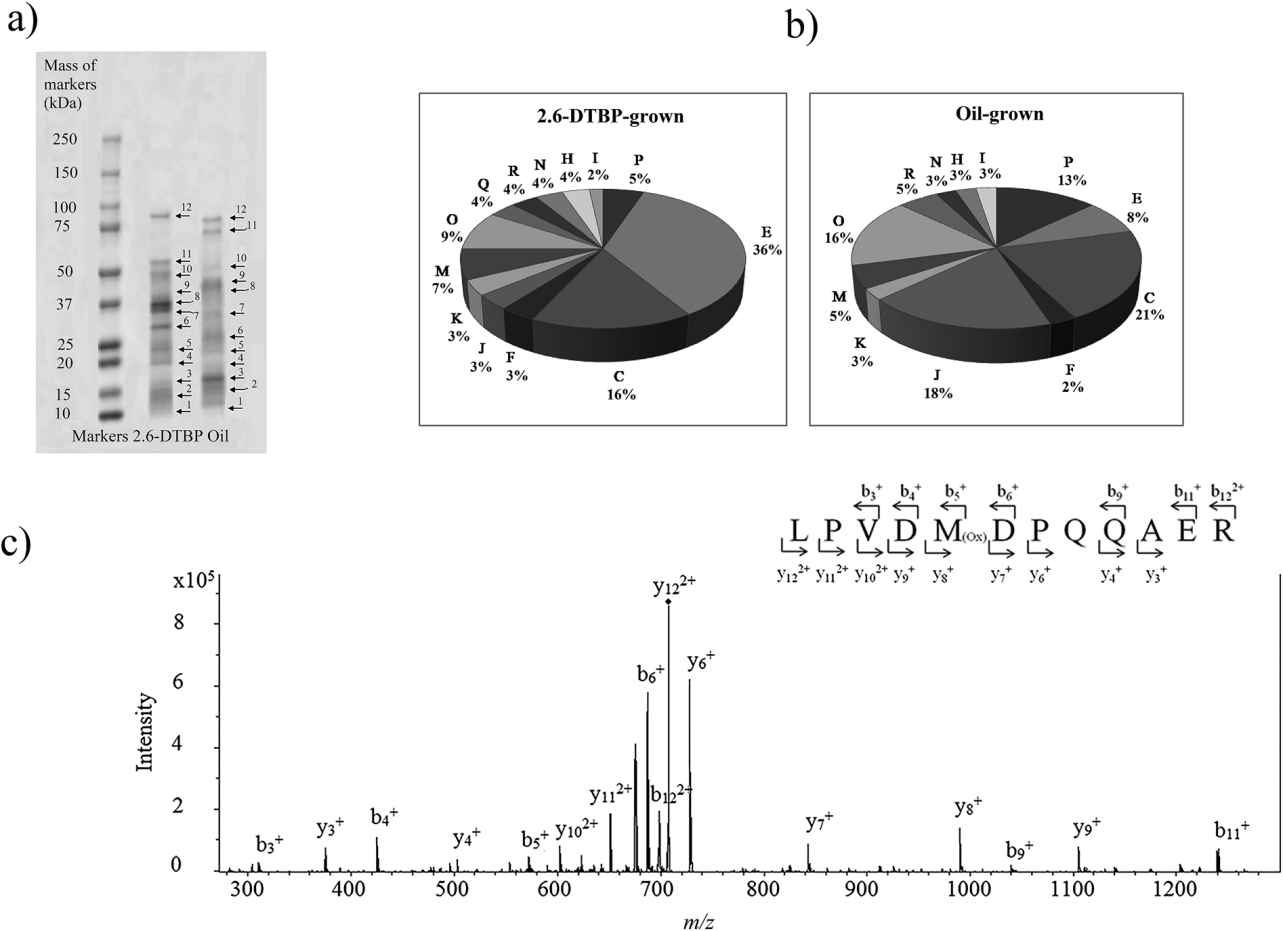
(a) SDS-PAGE electrophoresis of *P. aeruginosa* san ai proteins with the corresponding bonds (spot no. 1–12 for nanoLC-MS/MS analysis). (b) Functional classification of the proteins identified from *P. aeruginosa* san ai grown in MSM supplemented with 2,6-DTBP and oil. Metabolic categories. COG: C energy production; E: amino acid metabolism; I: lipid metabolism; J: translation; H: coenzyme metabolism; K: transcription; M: cell wall biogenesis; N: cell motility; O: PTM chaperon function; P: inorganic ion transport; R: general function prediction. COG categories: https://www.ncbi.nlm.nih.gov/COG/. (c) Tandem mass spectrum of LPVDMDPQQAER peptide from muconolactone δ-isomerase (*m*/*z* = 707.80). Proteins were analyzed by the proteomic method. Muconolactone δ-isomerase was identified by MASCOT search with a score of 90. Specific b and y ions were observed within the measured mass accuracy of 44 ppm. More than 30% of the fragment ions were identified to belong to the assigned peptide.

Proteins from cultures grown on two different substrates, namely, 2,6-DTBP *versus* sunflower oil, were separated by SDS PAGE (Fig. 4a), digested by trypsin, and analyzed using nanoLC-MS/MS. A list of differentially regulated proteins identified in 2,6-DTBP- *versus* oil-supplemented media during the growth of *P. aeruginosa* san ai is provided in Table 1. A total of 86 proteins were identified, out of which 43 and 29 were uniquely identified in 2,6-DTBP- and oil-amended cultures, respectively.

**Table 1:** Proteins identified in 2,6-DTBP-supplemented medium *versus* oil-supplemented minimal medium during the growth of *P. aeruginosa* san ai

Identified protein	Entry name	Mascot score	Peptide matched	Coverage (%)	Molecular mass (kDa)	Spot no	COG	Fold change^a^
Azurin	AZUR_PSEAE	1191	10	60	16	2^b^	C	+1.1
Azurin	AZUR_PSEAI	696	12	76	13.9	1	C	Absence
ATP synthase subunit alpha	ATPA_PSEAB	81	4	8	55.5	11	C	Presence
ATP synthase subunit beta	ATPB_PSEAB	78	3	9	49.5	10	C	−1.2
ATP synthase gamma chain	ATPG_PSEAB	85	2	6	31.5	6	C	Presence
Isocitrate lyase	ACEA_PSEAE	76	2	5	58.9	11	C	Presence
Isocitrate dehydrogenase [NADP]	IDH_PSEAB	118	4	10	45.6	9	C	Absence
Electron transfer flavoprotein subunit alpha	ETFA_PSEAE	46	1	3	31.4	7	C	Absence
Electron transfer flavoprotein subunit beta	ETFB_PSEAB	65	2	11	26.4	6	C	Absence
C4-dicarboxylate-binding periplasmic protein DctP	DCTP_PSEAE	176	6	22	37	9	C	Presence
Succinate-CoA ligase [ADP-forming] subunit alpha	SUCD_PSEAE	57	1	4	30.2	6	C	Absence
Dihydrolipoyl dehydrogenase	DLDH1_PSEAE	190	6	17	48.6	10	C	−1.2
Glycerol kinase 2	GLPK2_PSEAE	185	6	14	55.9	11	C	Presence
Cytochrome c4	CYC4_PSEAE	132	2	15	20.7	4	C	Presence
Glutarate-semialdehyde dehydrogenase DavD	DAVD_PSEAE	76	2	6	51.6	10	E	Presence
Glycine dehydrogenase	GCSP1_PSEAE	702	11	12	103.9	12	E	Presence
Glycine cleavage system H protein 1	GCSH1_PSEAE	48	1	7	13.8	1	E	Absence
Glutaminase-asparaginase	ASPQ_PSEAE	177	6	18	38.6	8	E	Presence
Putrescine-binding periplasmic protein SpuD	SPUD_PSEAB	132	5	16	40.6	9	E	+8.1
Spermidine-binding periplasmic protein SpuE	SPUE_PSEAE	54	1	5	40	9	E	Presence
Aromatic-amino-acid aminotransferase	PHHC_PSEAE	142	7	22	43.2	9	E	Presence
Leucine-, isoleucine-, valine-, threonine-, and alanine-binding protein	BRAC_PSEAE	128	2	6	39.7	9	E	+8.1
Aliphatic amidase	AMIE_PSEAB	70	3	10	38.5	8	E	Presence
Histidine ammonia-lyase	HUTH_PSEAB	56	1	2	53.7	10	E	Presence
Urocanate hydratase	HUTU_PSEA7	241	3	5	61.2	11	E	Presence
Aspartate aminotransferase	AAT_PSEAE	80	1	3	43.3	9	E	Presence
Branched-chain-amino-acid aminotransferase	ILVE_PSEAE	161	3	11	34.1	7	E	Presence
Ketol-acid reductoisomerase (NADP(+))	ILVC_PSEAB	164	4	17	36.4	7	E/H	Presence
Arginine deiminase	ARCA_PSEAE	215	5	15	46.4	9	E	Presence
Ornithine carbamoyltransferase, catabolic	OTCC_PSEAE	420	13	35	38.1	8	E	Presence
Argininosuccinate lyase	ARLY_PSEAB	99	4	11	51.6	10	E	Presence
*N*-Acetyl-gamma-glutamyl-phosphate reductase	ARGC_PSEAB	92	3	8	36.6	8	E	Presence
Methylmalonate-semialdehyde dehydrogenase [acylating]	MMSA_PSEAE	339	7	18	53.6	11	E	Presence
5-Aminovalerate aminotransferase DavT	DAVT_PSEAE	129	5	15	45.2	9	E	−1.2
*S*-Adenosylmethionine synthase	METK_PSEAB	78	2	6	42.7	9	E	Presence
Formate-dependent phosphoribosylglycinamide formyltransferase	PURT_PSEAB	47	2	6	42.3	9	F	Presence
Nucleoside diphosphate kinase	NDK_PSEAB	46	1	6	15.4	2	F	Absence
Orotate phosphoribosyltransferase	PYRE_PSEAB	83	1	8	23.3	4	F	Presence
6,7-Dimethyl-8-ribityllumazine synthase	RISB_PSEAB	85	2	19	16.4	2	H	Presence
Delta-aminolevulinic acid dehydratase	HEM2_PSEAE	167	6	21	37	8	H	Presence
Acyl carrier protein 1	ACP1_PSEAE	78	3	32	8.7	1	I	Absence
Acetyl-CoA acetyltransferase	ATOB_PSEAE	135	4	11	40.4	9	I	Presence
Lipid A deacylase PagL	PAGL_PSEAE	48	1	5	18.4	3	I	Absence
Isoleucine-tRNA ligase	SYI_PSEAB	60	1	1	105.4	12	J	Presence
Glutamyl-tRNA(Gln) amidotransferase subunit A	GATA_PSEAE	164	2	5	51.8	10	J	Presence
Ribosome-recycling factor	RRF_PSEAB	56	1	5	20.5	4	J	Absence
30S ribosomal protein S7	RS7_PSE14	57	2	15	17.6	3	J	Absence
50S ribosomal protein L21	RL21_PSEA8	44	1	10	11.7	1	J	Absence
30S ribosomal protein S16	RS16_PSEA7	61	2	33	9.2	1	J	Absence
30S ribosomal protein S9	RS9_PSEA7	44	1	6	14.6	2	J	Absence
50S ribosomal protein L20	RL20_PSEA7	44	1	8	13.3	1	J	Absence
50S ribosomal protein L25	RL25_PSEA8	164	4	27	21.9	4	J	Absence
Elongation factor Ts	EFTS_PSEAB	77	3	16	30.6	6	K	Presence
Elongation factor Tu	EFTU_PSEAB	86	2	5	43.3	9	K	Presence
DNA-binding protein HU-beta	DBHB_PSEAE	122	2	33	9.1	1	K	Absence
Outer membrane porin F	PORF_PSEAE	161	5	16	37.6	8	M	Absence
Outer membrane protein OprJ	OPRJ_PSEAE	85	2	5	51.9	10	M	Presence
Outer membrane protein OprM	OPRM_PSEAE	48	1	2	52.6	10	M	Presence
Outer-membrane lipoprotein carrier protein	LOLA_PSEAB	74	1	6	23.1	5	M	Presence
Protein TolB	TOLB_PSEAE	80	1	2	47.7	10	M	Presence
Porin D	PORD_PSEAE	114	5	10	48.4	9	M	Absence
Porin B	PORB_PSEAE	123	7	16	50.8	10	M	Absence
A-type flagellin	FLICA_PSEAI	622	8	23	40	8,12	N	+6.5
Glutathione hydrolase proenzyme	GGT_PSEAE	72	2	4	58.9	11	O	Presence
60 kDa chaperonin	CH60_PSEA7	183	4	10	57	11	O	−9.1
Chaperone SurA	SURA_PSEAE	48	1	3	46.9	10	O	Presence
Thiol : disulfide interchange protein DsbA	DSBA_PSEAB	894	7	34	23.4	5	O	+1.22
Thiol peroxidase	TPX_PSEAE	46	1	6	17.2	2	O	Absence
Thioredoxin	THIO_PSEAE	50	1	11	11.9	1	O	Absence
Alkyl hydroperoxide reductase C	AHPC_PSEAB	78	3	17	20.5	4	O	Presence
Chaperone protein DnaK	DNAK_PSEAB	64	2	3	68.4	10	O	Absence
10 kDa chaperonin	CH10_PSEU5	64	2	18	10.3	1	O	Absence
Bacterioferritin	BFR_PSEAE	66	1	5	17.9	2	P	Absence
Catalase	CATA_PSEAE	76	1	2	55.6	11	P	Presence
Fe(3+)-pyochelin receptor	FPTA_PSEAE	82	3	4	79.9	11	P	+1.1
Ferric uptake regulation protein	FUR_PSEAE	59	1	9	15.2	2	P	Absence
Mercuric transport protein periplasmic component	MERP_PSEFL	76	2	19	9.5	1	P	Absence
Superoxide dismutase [Fe]	SODF_PSEAE	50	1	5	21.3	4	P	1.0
Phosphate-binding protein PstS	PSTS_PSEAB	98	2	8	34.5	7	P	Absence
Muconolactone delta-isomerase	CATC_PSEAE	90	1	8	11.3	1	Q	Presence
Toluene-4-monooxygenase	TMOA_PSEME	61	2	9	58.1	11	Q	Presence
N5-carboxyaminoimidazole ribonucleotide mutase	PURE_PSEAE	138	2	19	16.9	3	R	Presence
Ecotin	ECOT_PSEAB	55	1	7	17.3	3	R	+1.6
UPF0312 protein PLES_04211	Y421_PSEA8	2659	9	52	20.8	4	S	1.0
Uncharacterized protein PA3922	Y3922_PSEAE	101	3	8	51.2	10	S	Presence
Uncharacterized protein PA1579	Y1579_PSEAE	60	1	6	22.1	4	S	+1.1

a Fold change in relative abundance is the ratio of the abundance of proteins between DTBP-grown *versus* oil-grown cultures from three biological replicates. + are upregulated and – are downregulated proteins. The absence of proteins is identified in oil-grown cultures, but not in DTBP-grown cultures.

b The same bond number in both the gels (proteins from oil- and DTBP-grown cells).

Muconolactone δ-isomerase (CATC_PSEAE) (Fig. 4a and c), a unique enzyme that contributes toward the aromatic *ortho*-degradation *via* catechol, and acetyl-CoA-acyltransferase, which is responsible for β-ketoadipate transformation into succinyl, were differentially upregulated (Fig. 3a). CATC was not found in the oil-grown culture, implying it's strong downregulation. In accordance with the upregulation of the *ortho*-pathway, which was determined by the proteomic analysis, ring cleavage reactions were analyzed by the enzymatic tests. As ring fission is oxygen-consuming, the enzyme activity of crude protein extracts on 2,6-DTBP was measured by an oxygen electrode. The enzyme extract of *P. aeruginosa* san ai grown on LB and 2,6-DTBP exhibited specific activities of 0.02 and 0.23 U mg^−1^, respectively, indicating the aromatic ring processing reaction, as well as implying the inducibility of enzyme(s) responsible for degradation. As suggested in the earlier studies regarding the biodegradation of alkylphenols using *ortho*- or *meta*-aromatic pathways *via* alkylcatechols,^14,15^ we investigated the ring cleavage enzyme activities on several aromatic compounds, namely, benzoate, catechol, and 2,6-DTBP, using the biomass of *P. aeruginosa* san ai grown on different C sources (Table 2). Catechol *ortho*-cleavage as a result of C12O activity was monitored as an increase in absorbance at 260 nm (indicating the accumulation of *cis*,*cis*-muconic acid),^34^ at 290 nm (implying the presence of 2,4-dialkyl-3-hydroxy-*cis*,*cis*-muconic acid, which was proposed to be the product of 2,6-DTBP degradation (Fig. 3b) (bathochromic shift of OH– group is approximately 30 nm)),^49^ and *meta*-cleavage was measured at 375 nm (accumulation of 2-hydroxymuconic semialdehydes).^33,34^ As shown in Table 2, our results revealed that all the tested substrates were preferentially *ortho*-cleaved. The catechol branch of the β-ketoadipate pathway was found to be preferred for several molecules: nitroaromatics, phenol, toluene, and benzene, while protocatechuate was derived from lignin monomers and chlorinated aromatics.^5^ Our results are in agreement with the *ortho*-cleavage of catechol and sodium benzoate by *P. putida*^50^ and *P. aeruginosa* 142.^51–53^ Furthermore, the *ortho*-degradation of catechol by crude enzyme preparation from biomass grown on 2,6-DTBP implies that 2,6-DTBP could be *ortho*-converted by the same enzyme to the derivative of *cis*,*cis*-muconic acid. The upregulation of CATC_PSEAE along with the enzyme activity of C12O supports the hypothesis that the biodegradation of 2,6-DTPB might be passing through the *ortho*-cleavage of the ring. Large absorbance enhancement at 290 nm could imply the existence of 2,4-dialkyl-3-hydroxy-*cis*,*cis*-muconic acid as the product of the *ortho*-cleavage of 2,6-DTBP assuming that the previous hydroxylation was available at positions 3 and 4, a step that was not revealed in this study. The hydroxylation reaction can be a result of the monooxygenase or hydroxylating dioxygenase action that added hydroxyl groups,^54^ yielding an intermediate that is further cleaved by a dioxygenase.^55,56^ Our proteomic study revealed the upregulation of toluene-4-monooxygenase (TMOA), the enzyme responsible for the hydroxylation of the aromatic substrate. The relatively broad substrate specificity of TMOA has been reported;^28^ therefore, it can be considered to be a good candidate for the hydroxylation of aromatic 2,6-DTBP.

**Table 2:** Comparison of C12O and C23O activity of *P. aeruginosa* san ai grown on different C sources against different enzyme substrates

C-source	Substrate	Specific activity, U mg^−1^	
		*A*_260_	*A*_375_
Sodium benzoate	Sodium benzoate	0.160	0.002
	Catechol	0.150	0.010
	2,6-DTBP	0.110; 0.150^a^	0.008
2,6-DTBP	2,6-DTBP	0.100; 0.160^a^	0.004
	Catechol	0.080	<0.001
Peptone	Sodium benzoate	0.020	<0.001
	2,6-DTBP	0.030	<0.001
	Catechol	0.009	0.007

a Absorbance determined at 290 nm only for 2,6-DTBP.

### Mechanisms of *P. aeruginosa* san ai adaptation to the aromatic substrate

**Fig. 5:**
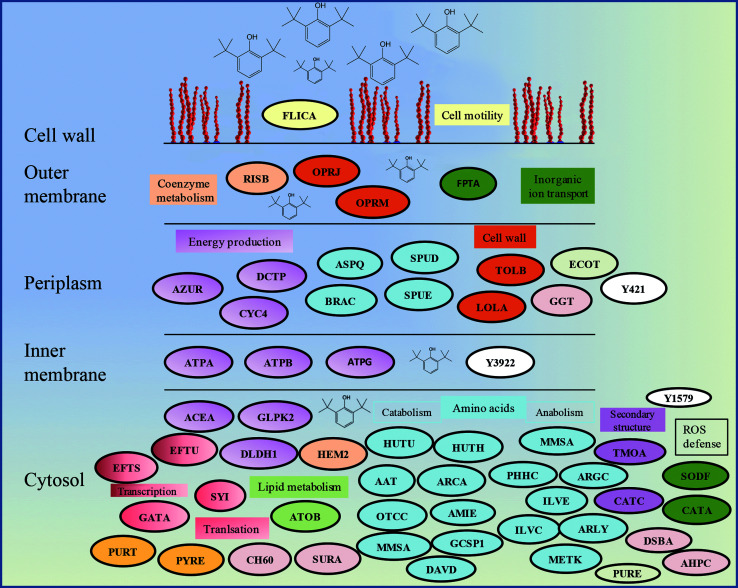
Schematic model of the response of *P. aeruginosa* san ai to the 2,6-DTBP plastic additive. Protein abbreviations are the same as that in Table 1. Proteins that belong to the same COG are labeled by the same color.

The proteomics analysis of *P. aeruginosa* san ai indicated that the core molecular responses to 2,6-DTBP can be summarized as the upregulation of proteins responsible for amino acid metabolism and energy production, while the translation, energy production, and inorganic ion transport and metabolism dominate in oil-amended cultures (Fig. 4b and 5).

#### Energy depletion

Energy production in the conditions of exposure to toxic substances is usually enhanced, and necessary proteins are upregulated as reported earlier.^19,23^ Our study shows that microorganisms use a specific metabolic path to prevent energy consumption and carbon leakage. That is, the key enzyme isocitrate lyase and AceA (Fig. 4a) of the glyoxylate shunt (which promote energy depletion, prevent the production of free radicals, and maintain the cellular redox potential by diminishing the electron flux into respiration) were uniquely found in 2,6-DTBP-grown cells. The glyoxylate shunt is a two-step metabolic pathway (isocitrate lyase and malate synthase) that serves as an alternative to the TCA cycle.^57^ On the other hand, the presence of isocitrate dehydrogenase (IDH)—an enzyme that explicitly transforms isocitrate to α-ketoglutarate—in the oil-amended culture suggests the existence of the complete Krebs cycle, instead of shunting as observed in the DTBP-grown culture. In fact, isocitrate is the branching point in the TCA, where shunting occurs or the complete cycle performs further. The enzymes of the glyoxylate shunt detected in this study showed that *P. aeruginosa* san ai rationalized the energy consumption and diminished the loss of carbon in the form of CO_2_ during the biodegradation of hydrocarbons. This idea is supported by the upregulation of dihydrolipoyl dehydrogenase (DLDH1) in the oil-grown culture. Namely, the activity of DLDH1 as a component of the α-keto acid dehydrogenase complex yields NADH and provides electrons for the respiratory chain, intensifying respiration, whereas the glyoxylate shunt reduces electron flux and respiration. The enzymes of glyoxylate bypass have been found in *Arthrobacter phenanthrenivorans* grown on phenantrene,^20^*A. chlorophenolicus* on phenol-grown cells,^58^ and *Mycobacterium gilvum* PYR-GCK on pyrene.^59^

#### Amino acid metabolism

An intensive amino acid metabolism reaction took place when *P. aeruginosa* san ai was grown on 2,6-DTBP (Fig. 4b and 5), similar to that when *P. putida* S-12 was grown on *p*-hydroxybenzoate. Remarkably, metabolism and the transport of branched, nonpolar amino acids were found to be upregulated. Several enzymes involved in the synthesis of nonpolar amino acids were identified: branched-chain amino acid aminotransferase (ILVE); ketol-acid reductoisomerase (NADP(+)); ILVC; methylmalonate semialdehyde dehydrogenase (MMSA); glutaminase asparaginase (ASPQ); aspartate aminotransferase (AAT); aromatic amino acid aminotransferase (PHHC) and its emphasized transport by high-affinity transport system I; and leucine-, isoleucine-, valine-, threonine-, and alanine-binding proteins (BRAC proteins). Apart from the anabolisms of nonpolar amino acids, the catabolism reactions of glycine, histidine, and arginine were intensified. The degradation of arginine through the arginine deiminase (ADI) pathway is a nonredox process that produces 1 mol of ATP and can be induced under various stress conditions.^60,61^ ADI enzymes are identified as upregulated in this study: arginine deiminase (ARCA) and catabolic ornithine carbamoyltransferase (OTCC). OTCC catalyzes the phosphorolysis of citrulline, yielding ornithine and carbamoyl phosphate that serves to generate ATP from ADP. At the same time, the enzymes of l-arginine biosynthesis, namely, *N*-acetyl-gamma-glutamyl-phosphate reductase (ARGC) and argininosuccinate lyase (ARLY), were identified, too. More than one-third of the upregulated proteins involved in amino acid metabolism were related to glutamine and glutarate metabolism: 5-aminovalerate aminotransferase DavT (DAVD), ASPQ, PHHC, histidine ammonia-lyase (HUTH), and AAT. A similar trend was reported by Vandera *et al.*^20^ The enzymes of the catabolic degradation of l-histidine (HUTH and HUTU) and glycine, namely, glycine dehydrogenase (GCSP1), were identified in the DTBP culture. GCSP1 is a part of the glycine cleavage system, (GCS) that is highly sensitive toward the alterations in the oxidation–reduction state of the respiratory chain (*e.g.*, oxidizing conditions stimulate while reducing conditions strongly inhibit this process). The intermediate of glycine cleavage, namely, *N*^5^,*N*^10^-methylene-H_4_ folate, can be used for the biosynthesis of *S*-adenosyl-methionine; this biosynthesis (*S*-adenosylmethionine synthase, METK) was upregulated. Histidine degradation yields glutamate, which can be further used for biosynthesis. Generally, the catabolism of glycine and histidine can be considered in relation to improved energy production and biosynthesis of basic building blocks for other structural molecules.

### Cell membrane proteins

It seems that the most massive changes occurred in the membrane when *P. aeruginosa* san ai was grown in the presence of DTBP *versus* oil as the C sources. In the DTBP-grown culture, the outer membrane OPRJ and OPRM proteins are differentially expressed, while porines D and B (PORD and PORB, respectively) are found in the oil-grown culture (Fig. 5). The overexpression of the channel-forming component of a multidrug resistance efflux pump OPRJ and OPRM— the major efflux pump for *n*-hexane and *p*-xylene—indicates the active response of the cell toward exposure to aromatic hydrocarbons; this is in good agreement with the fact that *P. aeruginosa* san ai can resist and grow when exposed to 2,6-DTBP. The increased content of the protein subunits of the solvent efflux pump systems and decreased contents of porins was registered in *P. putida* KT2440 exposed to phenol.^22^ An energized inner membrane is required for efflux that is in accordance with the upregulation of ATP synthase; this implies an increased production and consumption of ATP that can be partially used for efflux. Moreover, the identified protein Tol B (TOLB) and outer-membrane lipoprotein carrier protein (LOLA) are important for membrane integrity. TOLB from the Tol Pal system links the inner and outer membranes and the peptidoglycan layer, while LOLA acts in the translocation of lipoproteins from the inner membrane to the outer membrane. In accordance with the already mentioned chaperone SurA (SURA), which is involved in the correct assembly of the outer membrane proteins, OPRJ, OPRM, and TOLB show an adjustment of the membrane structure for the purpose of cell survival when exposed to 2,6-DTBP. At the same time, PORD and PORB reveal that an intensive diffusion of nutritive compounds, such as glycerol (PORB) and amino acids (PORD), occurs in the oil-grown culture. The enhanced glycerol amount can be attributed to the action of extracellular lipase from *P. aeruginosa* san ai that hydrolyzes oil to glycerol and fatty acids.^45^

#### Posttranslational modifications and chaperons

Our results reveal an overexpression of the translation elongation factors EFTU and EFTS and ribosomal proteins (GATA and RRF), which contribute toward protein translation, in cells grown in the DTBP-supplemented medium. On the other hand, in the oil-grown culture, several small ribosomal proteins and chaperons (CH60, CH10, and DNAK) were upregulated, indicating increased protein synthesis accompanied by protein processing. In the DTBP-grown culture, however, SURA, which is involved in the correct folding and assembly of the outer membrane proteins and is sensitive toward aromatic residues, was uniquely identified.

Posttranslational modifications are mainly related to protein redox processing: glutathione hydrolase proenzyme (GGT), thiol : disulfide interchange protein DsbA (DSBA), and alkyl hydroperoxide reductase C (AHPC) in DTBP cultures and thiol peroxidase (TPX) and thioredoxin (THIO) in oil-amended cultures (Table 1). As the mechanism of toxicity of phenol derivatives involves the production of reactive oxygen species (ROS) (*e.g.*, superoxide, hydroxyl radicals, hydrogen peroxide, and free radicals), enzymes that can neutralize ROS were identified as differentially expressed in the current study, such as catalase (catA) and AHPC, which are kinetically more potent H_2_O_2_ scavengers than CAT, as well as the fact that they are probably the primary scavengers of endogenous H_2_O_2_ (Fig. 5). Proteomic data were validated in the enzymatic study, revealing a slight increase in the SOD and even six times higher CAT-specific activity in *P. aeruginosa* san ai grown on 2,6-DTBP *versus* oil, confirming the oxidative stress caused by 2,6-DTPB (Table 3). Furthermore, the upregulation of DSBA responsible for SH-group processing revealed that the emphasized thiol–disulfide exchange was impacted by DTBP, too. In oil-amended cultures, thioredoxin that catalyzes dithiol–disulfide exchange reactions and thiol-specific peroxidase that catalyzes the reduction of hydrogen peroxide and organic hydroperoxides to water and alcohols were identified. Our results imply an efficient ROS defense and redox homeostasis maintenance in *P. aeruginosa* san ai cells exposed to both DTBP and oil.

**Table 3:** Superoxide dismutase and CAT activity of *P. aeruginosa* san ai grown on different C sources

C-source	Enzyme activities	
	SOD, U mg^−1^	Catalase, U mg^−1^
2,6-DTBP	18.7	2.68
Oil	9.51	0.40

### Degradation of 2,6-DTBP by crude enzyme preparation

Crude enzyme preparation obtained from *P. aeruginosa* san ai was investigated to degrade 2,6-DTBP (concentration: 10 mg mL^−1^). The crude enzyme preparation obtained from *P. aeruginosa* san ai grown on 2,6-DTBP, quickly within four days, degraded 98% of 2,6-DTBP (concentration: 10 mg L^−1^). A similar strategy used toward hydrocarbon removal was successfully applied by Kadri *et al.*^62^ They reported that crude enzymes extracted from *Alcanivorax borkumensis* showed high efficiency in terms of hydrocarbon removal. This approach demonstrated the better capacity of crude enzyme preparation in the bioremediation of aromatic pollutants as compared to using whole microbial cells. Accordingly, our results show that crude enzymes from *P. aeruginosa* san ai degraded 2,6-DTBP (concentration: 10 mg L^−1^) really rapidly in a shorter time (4 days) at almost the same efficiency as that obtained when using microorganisms for biodegradation (7 days), namely, 98 and 100%, respectively.

## 4. Conclusion

The effects of plastics, particularly microplastics, on aquatic environments and their biota have become a growing problem on our planet. Therefore, 2,6-di-*tert*-butylphenol as a plastic additive has been considered to be a serious issue for aquatic animals. This study was undertaken to estimate the capacity for 2,6-DTBP biodegradation by using the widespread *Pseudomonas* bacteria and the effects that it causes on these microorganisms. *P. aeruginosa* san ai rapidly degraded 2,6-DTBP during the growth in a mineral medium that mimics the aquatic environment. A high degradation capacity of 85% out of 100 mg L^−1^ of 2,6-DTBP for 7 days was determined. The removal of 2,6-DTBP completely dissolved in water (4 mg L^−1^) by *P. aeruginosa* san ai was almost accomplished within 10 h, causing intensive respiration. Further, it has been shown that crude enzymes isolated from *P. aeruginosa* san ai grown on 2,6-DTBP as the sole C source degraded 2,6-DTBP rapidly in a shorter time with higher efficiency than those obtained when using whole cells.

A simple strategy, based on one-dimension electrophoresis of the total protein extracts followed by proteomic analysis (nanoLC-MS/MS), revealed that the proteins that were upregulated in the presence of 2,6-DTBP could be assigned to the functional groups of energy production and amino acid metabolism. As a response to the exposure to 2,6-DTBP, the culture shifts into a glyoxylate shunt to save energy and carbon. Further, at the cell membrane level, the content of solvent efflux pump systems increases and that of porines decreases, implying a large change in the membrane structure. Proteins responsible for redox homeostasis are upregulated in both DTBP- and oil-amended cultures, but enzymatic tests have shown more intensive oxidative stress in DTBP-amended cultures. Genomic, enzymatic, and proteomic data accordingly revealed that 2,6-DTBP could be *ortho*-cleaved. All these mechanistic insights related to catabolism and microbial adaptation to 2,6-DTBP may have an impact on the biodegradation, bioremediation, and biocatalysis by *P*. *aeruginosa* san ai. Finally, although *ortho*-disubstituted phenols are complex molecules for biodegradation, the *P. aeruginosa* san ai strain holds promising potential for the biotransformation of 2,6-DTBP; as such, the strain could be a promising agent in the bioremediation of the environment, particularly water, contaminated with organic pollutants.

The multiomics approach based on a combination of genomics and proteomics has provided insights into the global metabolic and regulatory networks of *P. aeruginosa* san ai that are exposed to 2,6-DTBP. While the genome of *P. aeruginosa* san ai revealed the existence of genes responsible for proteins that could serve for microorganism adaptation, a comparative proteomic analysis enabled the profiling of up- and downregulated proteins involved in the metabolic control of microorganisms grown on different C sources. As a result, the proteomic study gave an overall understanding of the response mechanisms toward pollutants and an opportunity to selectively use induced core proteins for environmental biomonitoring in natural microbial communities exposed to organic pollutants. The sets of up- or downregulated proteins obtained by proteomics might be used as multi-markers toward environmental contamination. Ultimately, *P. aeruginosa* san ai can be used for designing new strategies for environmental protection, as well as for the engineering of novel strains and novel proteins with improved characteristics for more effective bioremediation of contaminated areas.

## Conflicts of interest

There are no conflicts to declare.

## Supplementary Material

RA-009-C9RA04298A-s001

## References

[cit1] OECD/SIDS , Screening Information Data Set (SIDS) of OECD High Production Volume Chemicals Programme, 1994

[cit2] Catalog Plastic Additive Standards Guide, New Haven, USA, 2018, https://www.accustandard.com/plastic-additive-catalog-2nd-edition

[cit3] Rezania S., Park J., Md Din M., Mat Taib S., Talaiekhozani A., Yadav K., Kamya H. (2018). Mar. Pollut. Bull..

[cit4] Vidali M. (2001). Pure Appl. Chem..

[cit5] Wells T., Ragauskas J. (2012). Trends Biotechnol..

[cit6] Krastanov A., Alexieva Z., Yemendzhiev H. (2013). Eng. Life Sci..

[cit7] Brzeszcz J., Kaszycki P. (2018). Biodegradation.

[cit8] Kwon K. H., Yeom S. H. (2009). Bioprocess Biosyst. Eng..

[cit9] Sridevi V., Chandana Lakshmi M. V. V., Manasa M., Sravani M. (2012). IJESAT.

[cit10] Jeong J. J., Kim J. H., Kim C., Hwang I., Lee K. (2013). Microbiology.

[cit11] Takeo M., Prabu S. K., Kitamura C., Hirai M., Takahashi H., Kato D., Negoro S. (2006). J. Biosci. Bioeng..

[cit12] Ajithkumar B., Ajithkumar V., Iriye R. (2003). Res. Microbiol..

[cit13] Kohler H. P., Maarel M., Kohler-Staub D. (1993). Appl. Environ. Microbiol..

[cit14] Toyama T., Maeda N., Murashita M., Chang Y., Kikuchi S. (2010). Biodegradation.

[cit15] Tuan N. N., Hsieh H. Ch., Lin Y. W., Huang Sh. L. (2011). Bioresour. Technol..

[cit16] Zhang Y., Fang Z., Xu D., Xiao Y., Zhao J., Qiang Z. (2005). J. Environ. Sci..

[cit17] Chauhan A., Jain R. K. (2010). Biodegradation.

[cit18] Kurbatov L., Albrecht D., Herrmann H., Petruschka L. (2006). Environ. Microbiol..

[cit19] Li F., Song W., Wei J., Liu C., Yu C. (2016). Int. Biodeterior. Biodegradation.

[cit20] Vandera E., Samiotaki M., Parapouli M., Panayotou G., Koukkou A. I. (2015). J. Proteomics.

[cit21] Verhoef S., Ballerstedt H., Volkers J. M. R., de Winde H. J., Ruijssenaars H. J. (2010). Appl. Microbiol. Biotechnol..

[cit22] Roma-Rodrigues C., Santos P. M., Benndorf D., Rapp E., Sá-Correia I. (2010). J. Proteomics.

[cit23] Santos M. P., Benndorf D., Sá-Correi I. (2004). Proteomics.

[cit24] Seo J. S., Keum Y. S., Li Q. X. (2009). Int. J. Environ. Res. Public Health.

[cit25] Tsirogianni I., Aivaliotis M., Karas M., Tsiotis G. (2004). Biochim. Biophys. Acta.

[cit26] Kahlon R. S. , Biodegradation and bioremediation of organic chemical pollutants by Pseudomonas, in Pseudomonas: Molecular and applied biology, ed. R. Kahlon, Springer International Publishing Switzerland, 2016. pp. 343–417

[cit27] Karadzic I., Masui A., Fujiwara N. (2004). J. Biosci. Bioeng..

[cit28] Tao X., Lu G., Liu J., Li T., Yang L. (2009). Int. J. Environ. Res. Public. Health.

[cit29] Lin M., Hu X., Chen W., Wang H., Wang C. (2014). Int. Biodeterior. Biodegradation.

[cit30] Heyd M., Kohnert A., Tan T. H., Nusser M., Kirschhöfer F., Brenner-Weiss G., Franzreb M., Berensmeier S. (2008). Anal. Bioanal. Chem..

[cit31] Rosenberg M., Gutnick D., Rosenberg E. (1980). FEMS Microbiol. Lett..

[cit32] Bradford M. M. (1976). Anal. Biochem..

[cit33] Briganti F., Pessione E., Giunta C., Scozzafava A. (1997). FEBS Lett..

[cit34] Mahiudddin Md., Fakhruddin A. N. M., Mahin A. A. (2012). ISRN Microbiol..

[cit35] Mars A., Kingma J., Kaschabek S., Reineke W., Janssen D. (1999). J. Bacteriol..

[cit36] Mancini S., Abicht H. K., Gonskikh Y., Solioz M. (2015). Mol. Microbiol..

[cit37] Sala-Trepat J. M., Evans W. C. (1971). Eur. J. Biochem..

[cit38] Roland F. B., Irwin W. S. (1952). J. Biol. Chem..

[cit39] Sun M., Zigman S. (1987). Anal. Biochem..

[cit40] Tribedi P., Sil A. K. (2013). J. Appl. Microbiol..

[cit41] Kadam T. A., Rupa L., Balhal D. K., Totewad N. D., Gyananath G. (2009). Asian J. Exp. Sci..

[cit42] Walker J. D. , Testing decisions of the TSCA Interagency Testing Committee for chemicals on the Canadian Environmental Protection Act domestic substances list and priority substances list: Di-*tert*-butylphenol, ethyl benzene, brominated flame retardants, phthalate esters, chloroparaffins, chlorinated benzenes and anilines, Environmental toxicity and risk assessment, ASTM, Philadelphia, 1996

[cit43] Zhang C., Zeng G., Yuan L., Yu J., Li J., Huang G., Xi B., Liu H. (2007). Chemosphere.

[cit44] Sasaki M., Maki J., Oshiman K., Matsumura Y., Tsuchido T. (2005). Biodegradation.

[cit45] Karadzic I., Masui A., Izrael-Zivkovic L., Fujiwara N. (2006). J. Biosci. Bioeng..

[cit46] Basha K. M., Rajendran A., Thangavelu V. (2010). Asian J. Exp. Biol. Sci..

[cit47] Freitag D., Ballhorn L., Geyer H., Korte F. (1985). Chemosphere.

[cit48] Izrael-Živković L., Beškoski V., Rikalović M., Kazazić S., Shapiro N., Woyke T., Gojgić-Cvijović G., Vrvić M., Maksimović N., Karadžić I. (2019). Extremophiles.

[cit49] Silverstain R. and Bassler G., Spectrometry Identification of Organic Compounds, John Wiley&Sons, Inc., New York, 1967

[cit50] Loh K., Chua S. (2002). Enzyme Microb. Technol..

[cit51] Feisti C. F., Hegeman G. D. (1969). J. Bacteriol..

[cit52] Haigler B. E., Pettigrew C. A., Spain J. C. (1992). Appl. Environ. Microbiol*.*.

[cit53] Romanov V., Hausinger R. P. (1994). J. Bacteriol..

[cit54] Karigar C. S., Rao S. S. (2011). Enzyme Res..

[cit55] Harayama S., Kok M., Neidle E. L. (1992). Annu. Rev. Microbiol..

[cit56] Vaillancourt F. H., Bolin J. T., Eltis L. D. (2006). Crit. Rev. Biochem. Mol. Biol..

[cit57] White D. , The Physiology and Biochemistry of Procariotes, Oxford University Press, New York, 2nd edn, 2000

[cit58] Unell M., Abraham P. E., Shah M., Zhang B., Rückert C., VerBerkmoes N. C. (2009). J. Proteome Res..

[cit59] Badejo A. C., Choi C. W., Badejo A. O., Shin K. H., Hyun J. H., Lee Y. G. (2013). Biodegradation.

[cit60] Eschbach M., Schreiber K., Trunk K., Buer J., Jahn D., Schobert M. (2004). J. Bacteriol..

[cit61] Izrael-Živković L., Rikalović M., Gojgić-Cvijović G., Kazazić S., Vrvić M., Brčeski I., Beškoski V., Lončarević B., Gopčević K., Karadžić I. (2018). RSC Adv..

[cit62] Kadri T., Magdouli S., Rouissi T., Kaur Brar S. (2018). Biochem. Eng. J..

