# Supplementary material for: A comprehensive study of conditions of the biodegradation of a plastic additive 2,6-di-tert-butylphenol and proteomic changes in the degrader Pseudomonas aeruginosa san ai
Source: RSC Adv. 2019 Jul 30;9(41):23696–710. doi: 10.1039/c9ra04298a (PMC9069449; doi:10.1039/c9ra04298a)
Supplement: RA-009-C9RA04298A-s001 [file RA-009-C9RA04298A-s001.pdf]

Electronic Supplementary Material (ESI) for RSC Advances.
This journal is © The Royal Society of Chemistry 2019

Electronic Supplementary Information

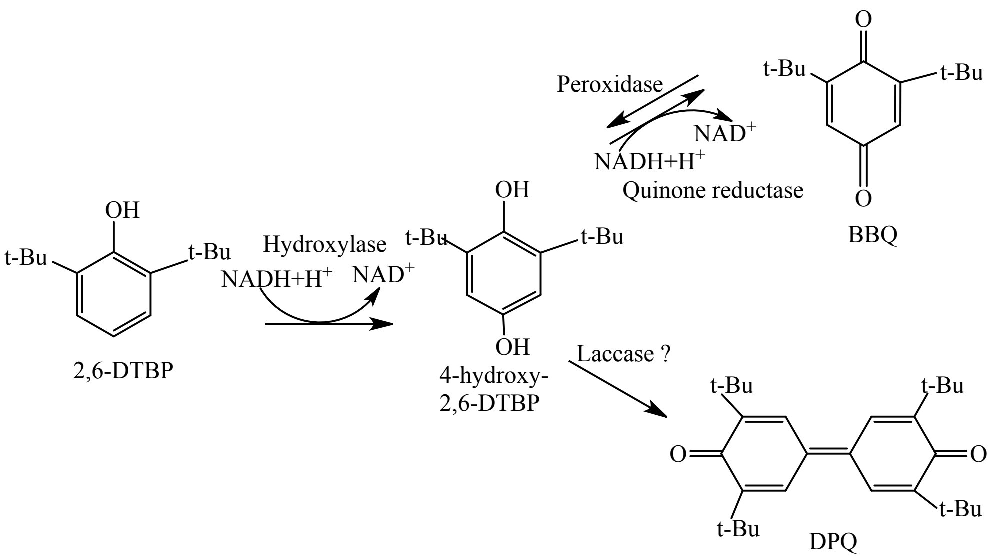

GC-MS analysis of degradation of high concentration of 2,6-DTBP (400 mg/L) confirmed the presence of 2,6-di-*tert*-butylbenzoquinone, BBQ (*m/z* 220) and 3,3,5,5-tetra-*tert*-butyl-4,4-diphenoquinone, DPQ (*m/z* 408), which, as quinone derivatives, are toxic for microorganism with EC_{50} of 0.5 mg/L (Walker 1988) and inhibited the pseudomonad at concentration of 10 mg/L (Trevors and Basaraba 1980). However, quinone and related with that their derivatives, could be reduced by NAD(P)H-quinone oxidoreductase (EC 1.6.5.5.) and further processed. Under the exposure to concentration of 400 mg/L of 2,6-DTBP *P. aeruginosa* san ai showed NAD(P)H-quinone oxidoreductase activity of 192 mU/mg. Both of identified metabolites- BBQ and DPQ could be reduced to hydroquinone derivatives whose toxicity is very low (Trevors and Basaraba 1980) and furthermore degraded as proposed for 2,6-DTBP.
